# Transanal fistulectomy for postoperative persistent rectourethral fistula in patients with ARM: is simple resection enough?

**DOI:** 10.1186/s12893-021-01186-3

**Published:** 2021-04-02

**Authors:** Xinjie Huang, Yajun Chen, Wenbo Pang, Chunhui Peng, Dongyang Wu

**Affiliations:** Department of General Surgery, Beijing Children’s Hospital, Capital Medical University, National Center for Children’s Health, No. 56 Nanlishi Road, Beijing, 100045 China

**Keywords:** Rectourethral fistula, Persistent fistula, Anorectal malformation, Surgical technique

## Abstract

**Background:**

Postoperative rectourethral fistula (RUF) in patients with congenital anorectal malformation (ARM) remains a challenge for paediatric surgeons, among them persistent fistula is the most common. Various techniques have been proposed, only a few reports based on different causes are available, and there is no consensus so far. This study is to evaluate the application, advantages and limitations of transanal fistulectomy approach in repairing persistent RUF in ARM patients.

**Methods:**

From January 2007 to July 2019, 78 ARM patients who received revisional surgery for RUF were reviewed, 34 persistent fistulas were identified. Examination under anaesthesia included patients with fistulas that were located within 3 cm from the anus verge, good appearance of the anus and sphincter function, and no urethral and rectoanal obstruction. Three patients were excluded because of complex urologic pathologic defects. In total, thirty-one patients underwent transanal fistulectomy to repair RUF.

**Results:**

All cases were approached with transanal incision and fistulectomy to repair RUF. The average operative time was 91 ± 35 min. At a minimum six-month follow-up, 29 patients healed after the first attempt, the success closure rate was 93.5%. Two patients received redo transanal fistulectomy and healed. Two patients had postoperative complications: one patient had urethral stenosis and it was managed by dilation; one patient had urethral diverticulum but it did not require revisional surgery. No patient in this study was incontinent because of the surgery.

**Conclusions:**

Transanal fistulectomy provides a simple, straightforward, and safe approach to repair persistent RUF in ARM patients, especially in those with a low-lying fistula, good anus appearance and sphincter function without obstruction in the rectum or urethra.

*Trial registration:* Retrospectively registered.

## Background

Postoperative rectourethral fistula (RUF) in congenital anorectal malformation (ARM) patients remains a challenging issue for paediatric surgeons. This complication has three forms: persistent, recurrent, and acquired types. Various surgical techniques have been proposed, but they are mainly based on a small number of cases, and recurrence is common [1–3]. Moreover, although there is a trend to emphasize the need for highly-individualized reoperation plans, only a few reports based on different causes are available [4].

In this study, we present our experience of the detailed procedures of transanal fistulectomy to repair postoperative persistent fistulas in ARM patients. The main purpose is to evaluate the application and advantages of this approach, as well as its limitations.

## Methods

Single-centre retrospective cases from January 2007 to July 2019 were reviewed at the Beijing Children’s Hospital Affiliated to Capital Medical University, National Children’s Health Center. The institutional review board approved this analysis. This retrospective observational study was conducted in accordance with the Strengthening the Reporting of Observational studies in Epidemiology (STROBE) guidelines. A total of 78 male ARM patients received revisional surgery for RUF during the study period. The original classification of ARM (Krickenbeck classification), previous surgery details, and reoperation findings were used to identify RUF’s aetiology.

### Preoperative work-up and surgical indication

**Fig. 1:**
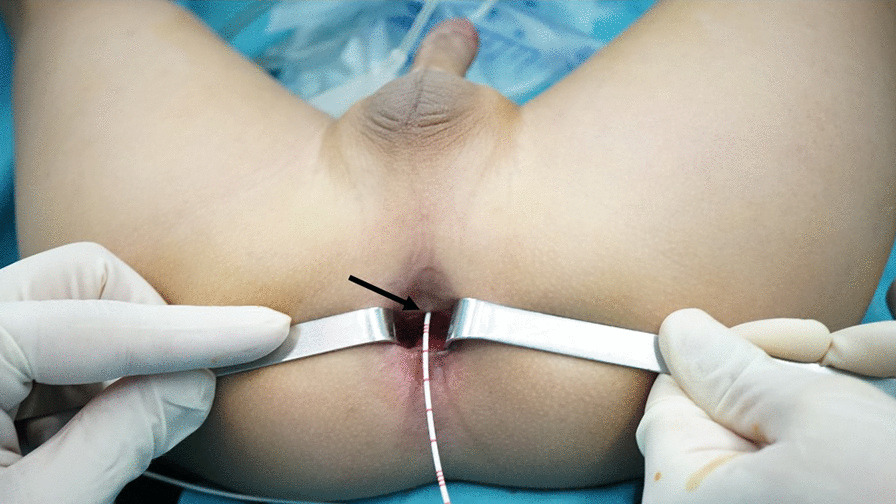
Examination under anaesthesia: the appearance of the anus and sphincter function were good. The fistula (arrow) was located 1 cm above the anus verge without obvious scar

Patients who had postoperative rectourethral fistulas after the major repair of ARM were the candidates for this surgical approach. Next, we examined patients under anesthesia, and we believed it was essential to establish the diagnosis, evaluate the fistula, and exclude the coexist rectoanal and/or urethral defects. An electricity stimulator was used to determine the location of the anus. Then, the fistula site, size was carefully measured. Persistent fistula was defined as congenital RUFs that were left intact in the primary anorectoplasty, and it was not supposed to be surrounded by dense scar. To perform transanal fistulectomy, the following conditions should be satisfied: (1) the location of the fistulas were within 3 cm from the anus verge; (2) good anus appearance and sphincter function; and (3) no urethral or rectoanal obstruction (Fig. 1).

### Perioperative managements

Bowel preparation started two weeks before the surgery. Patients were given liquid food and daily normal saline solution enema until they passed clear liquid freed of stool through the anus.

After the surgery, oral feeding was resumed after the bowel function returned in patients with faecal diversion; otherwise, the children abstained from food for at least 5 to 7 days and continued to receive total parenteral nutrition. The intravenous antibiotic continued for 7 days. The surgical site was kept clean and dry. The secretions and mucus were carefully wiped and cleaned frequently with sterile cotton. The Foley urethral catheter was removed at least 2 to 3 weeks postoperatively.

Follow-up was conducted in out-patient clinics or via telephone for at least 6 months. The successful closure was defined as the absence of symptoms and radiographic confirmation.

## Surgical technique

**Fig. 2:**
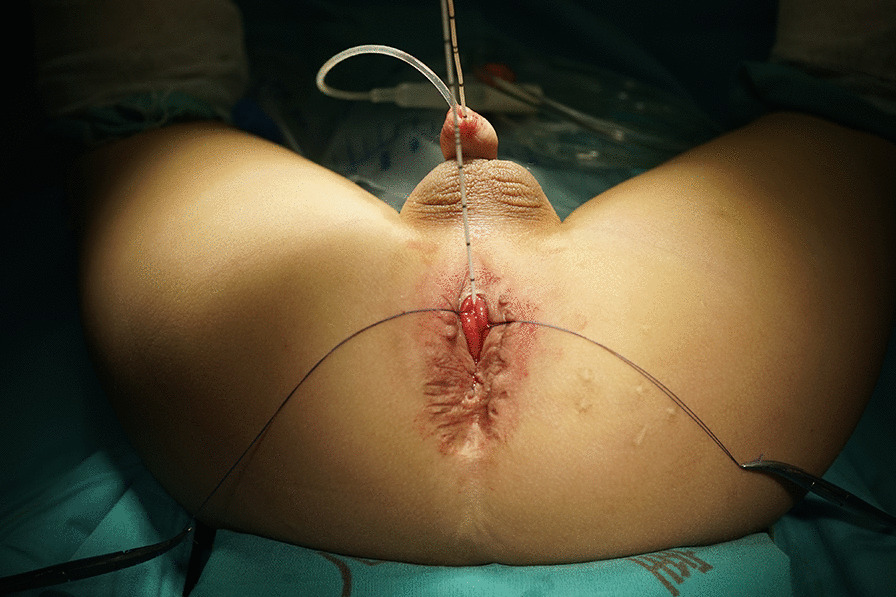
A Foley catheter in the urethra and a guide catheter in the fistula

The patient was placed in exaggerated lithotomy position with the pelvis elevated so that the anus and perineum were horizontal. A Foley catheter with an appropriate size that suits the patient was introduced into the bladder to avoid urethral damage. Another smaller catheter was introduced to enter the rectum through the fistula and served as a guide. We usually used an F3 urethral stent as the guide catheter (Fig. 2). Normally, an index finger can be easily placed inside the rectum; with the finger pressing the proximal urethra above the fistula, the guide catheter can go through the fistula without difficulty in patients with no anal stenosis. In small fistulas, the guide catheter hardly goes through the fistula, and injection of methylene blue solution via the Foley catheter helps to identify the rectal orifice.

**Fig. 3:**
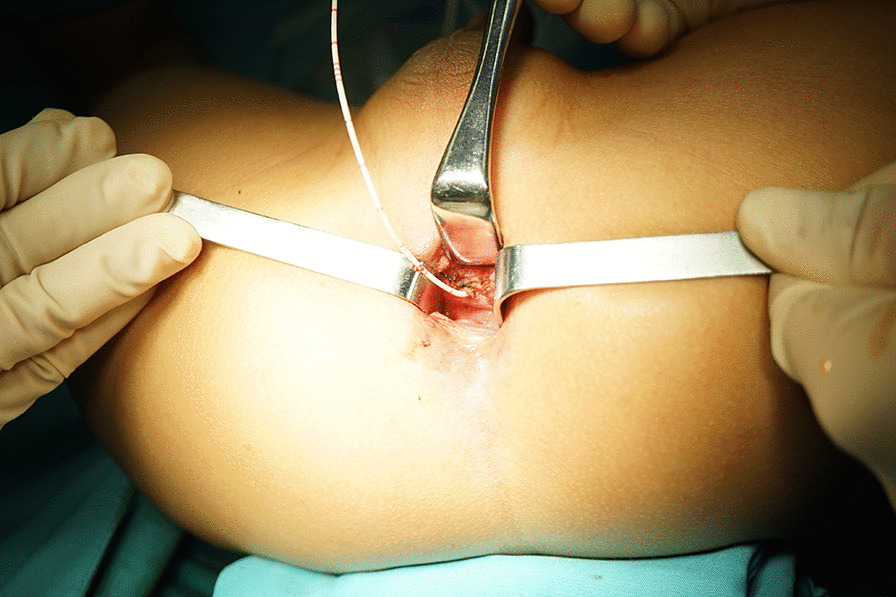
Minimal surgical incision provided enough exposure

An incision was made on the perineal body and the anterior anus verge, the surgeon must remain completely on the midline to open the sphincter mechanism. Normally, in the case of persistent fistula, the rectal orifice is close to the juncture of the rectal mucosa and skin. The incision extended longitudinally with the help of a retractor to gain enough exposure to the fistula (Fig. 3).

**Fig. 4:**
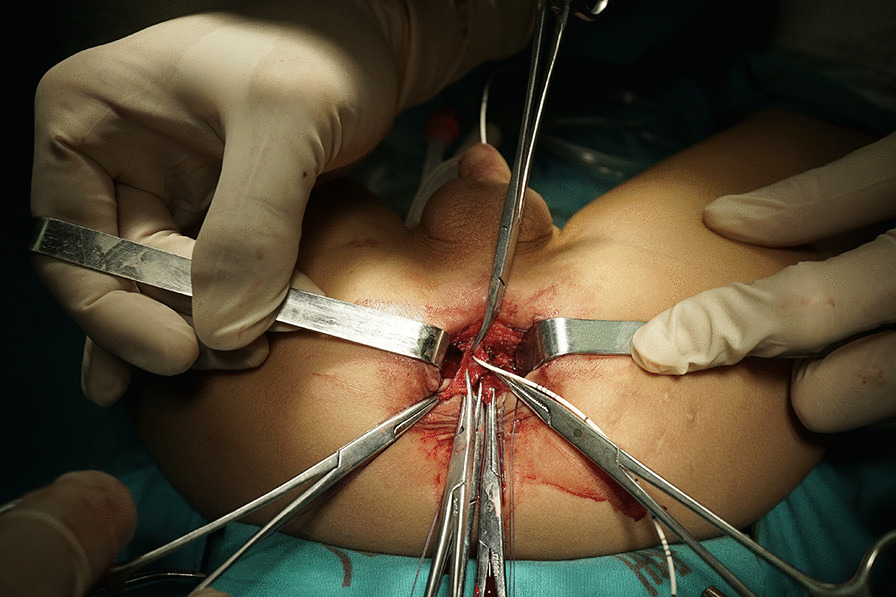
Forceps were distributed at the edge of the fistula to carry out uniform traction

At this point, an incision was made circumferentially approximately 1 mm away from the fistula margin on the rectal mucosa with an electric scalpel. 5–0 absorbable sutures or forceps were distributed at the edge of the fistula to carry out uniform traction (Fig. 4). Then, the fistula was freed from the surrounding tissue to the urinary tract. We used a scalpel or scissor to resect the fistula, avoiding the use of any electric system, to preserve the supporting blood supply around the surgical site.

**Fig. 5:**
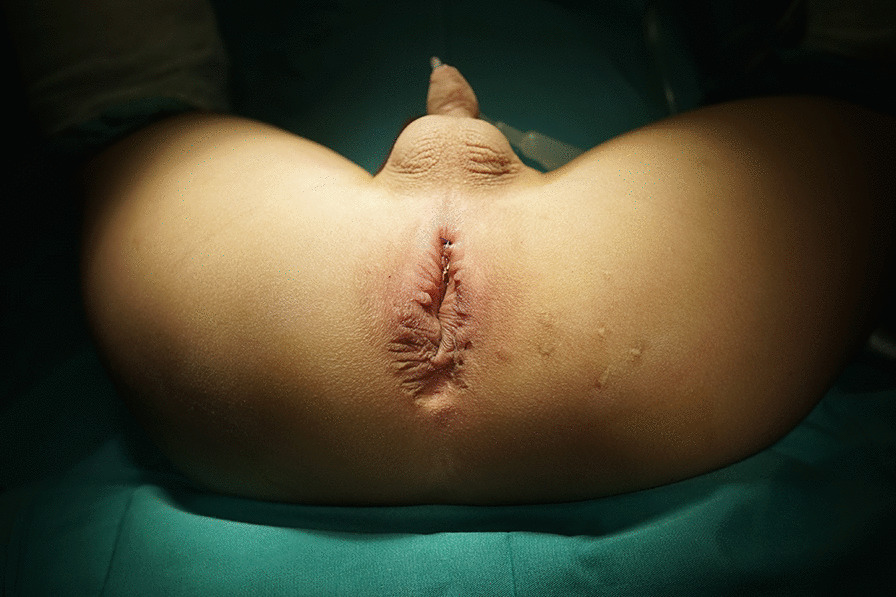
Final appearance after the repair and construction

Most fistulas are 4 to 5 mm long. When the Foley catheter was reached, the fistula was resected with scissors. The urethral defect was closed from inside the rectum by interrupted suture using 6–0 Vicryl. The rectum wall was closed longitudinally in single layer suture without tension. The final step was to reconstruct the levator ani muscle and the perineal body (Fig. 5).

## Results

Seventy-eight ARM patients who received reoperations for RUF were identified, and 34 persistent RUFs were included. Among them, 3 patients were excluded because of complex obstructed urethra on examination under anaesthesia. All 31 patients finally included were congenital rectourethral fistula, and they received one-stage anorectoplasty surgery during the neonatal period outside our centre. Thirty patients had anterior perineal anorectoplasty, and one patient had posterior sagittal approach. The average age at reoperation was 61 months (8–179 months). The average operative time was 91 ± 35 min, and all operations required no blood transfusion.

Twenty-nine patients healed after the first revisional surgery during the follow-up period. In two patients, the fistula recurred and healed after receiving a second revisional surgery at least six months after the first repair. There was no subsequent recurrence. The overall fistula closure rate at the first revisional surgery was 93.5% (29/31). Three patients had colostomy before transferred to us, and the remaining 28 patients did not undergo colostomy. None of the patients had invasive urinary diversion.

During a minimum 6-month follow-up, one patient had urethral stenosis and it was managed by routine urethral dilation. Another patient had urethral diverticulum, but it did not influence the preservation of urinary continence; he required no further surgical intervention. There were no reports of anal stenosis. No patient in this study was incontinent because of the surgery. The clinical characteristics of all patients are summarized in Table 1.

**Table 1:** Clinical characteristics of study patients

Characteristics	n = 31
Age (months)	60.9 ± 45.1
Primary anorectoplasty done elsewhere	
Anterior perineal anorectoplasty	30
Posterior sagittal approach	1
Fecal diversion	3
Invasive urinary diversion	0
Fistula site* (cm)	1.78 ± 0.79
Fistula size (mm)	5.1 ± 4.2
Operative time (min)	91 ± 35
Blood transfusion (ml)	0
Hospital stay (days)	23.1 ± 7.2
Success after the first attempt (%)	29 (93.5)
Complication	
Urethral stenosis	1
Urethral diverticulum	1

*Above the anus verge

## Discussion

In 1982, deVries and Pena [5] proposed posterior sagittal approach (PSARP) to repair ARM, a great advance has been achieved over the past 40 years since then. However, postoperative urologic complications that need subsequent revisional surgeries continue to exist [6]. Among them, persistent fistulas are the most common of all postoperative RUF cases [7]. In this retrospective study, all patients had one-stage anoplasty in the neonatal period in a general hospital, and most of them were treated by surgeons experienced with adult patients. We suspect that surgeons lack enough understanding of congenital RUF anatomy, and an old fashion invertogram showing a low-lying blind rectal pouch led them to perform anoplasty through the perineum area. The congenital RUFs were not identified and repaired, and thereby, patients had symptoms immediately after surgery. It is a preventable complication if the basic diagnostic and surgical principles about congenital ARM are followed. However, as other reports suggested, China still lacks enough well-trained paediatric surgeons [8]. It seems reasonable to recommend that patients go to tertiary hospitals to receive centralized treatments.

In making reoperation plan, we believe that the persistent fistula is different from acquired or recurrent RUF: (1) the fistula tract is left intact, and its surrounding tissue is elastic and flexible without scar or adhesion, which is suitable for healing; (2) the low-lying fistula opening in the rectum is easily exposed from below [9], most of which, in our study lies within 3 cm above the anus verge; this is consistent with the fact that most male RUF are bulbar fistula; (3) patients have a nearly “normal” appearing anus lying within the sphincter mechanism, it is plausible to only remove the fistula with limited incision. The three characteristics of the persistent RUF suggest that a simple and straightforward transanal fistulectomy without extensive incision and dissection is feasible. In our case series, 29 patients (93.5%) managed by this fashion had successful closure at the first attempt, with a relatively shorter operative time. The low recurrence is consistent with the current large case series using posterior sagittal approach to repair such fistulas [4]. Therefore, we suggest transanal fistulectomy as an alternative approach to repair the persistent RUF.

In terms of prognosis, reoperation for ARM is reported to have a less optimal function outcomes [10]. The biggest advantage of this approach is that it minimizes the interference with the continence mechanism. We avoided intensive incision on the ‘normal’ anus and used the guide catheter to stay close to the fistula. Technically, endorectal mucosectomy is sufficient to close the fistula [11]. We emphasize using scissors or scalpels to remove the fistula instead of electric ablation. In this way, we avoided any potential damage to the delicate transmural blood supply. None of our patients reported continence loss due to reoperation, indicates that this approach helps preserve continence.

Another advantage of this manoeuvre is to re-examine the anus postoperatively at 6 months without daily dilation, none of our patients had anal stenosis, which suggests that dilation can be safely avoided. This approach limits its incision on the anterior rectal wall, and subsequent scarring rarely affects the new anal size. This approach could also simplify the home care process for the patients, as well as minimize the trauma to the children.

In patients undergoing the transanal fistulectomy approach, a diverting colostomy may not be necessary. Our routine is to start adequate bowel preparation at least two weeks before reoperation to ensure that the patient passes clear liquid. Patients will fast food for least five days and have intensive and careful wound care daily. The Foley catheter stays at least two weeks postoperatively. The wound care plan aims at keeping the surgical site clean and dry, which has proven very useful in our other colorectal surgeries [12]. We did not observe any wound infection with or without colostomy during follow-up, which led us to believe that the colostomy can be avoided in such cases if basic rules are followed. However, if one is not confident about the suture and repair, he or she should not hesitate to divert.

We are also aware of some limitations in using this approach. The indication for this approach warrants thorough examination of the fistula, anus and urethra under anaesthesia. For fistulas located anywhere higher than 3 cm, surgeons may have to split the posterior rectal wall or adopt an additional abdominal approach to gain more exposure. Obstruction in the rectum or urethra puts extra pressure on suture line, endangering the healing process. For that reason, some authors suggested removing any obstruction before RUF repair [9]. Furthermore, a mislocated anus often requires extensive dissection to mobilize the rectum and relocate it, and this transanal approach may not fulfil this goal. Surgeons should evaluate the patients carefully and prepare for those complex situations, and this could only be done under anaesthesia. In our experience, if the indications mentioned above are fulfilled, the outcomes will be optimal.

## Conclusion

The transanal fistulectomy provides a simple, straightforward, and safe approach to repair persistent RUF in ARM patients, especially in those with a low-lying fistula, good anus appearance and sphincter function without obstruction in the rectum or urethra. This approach minimizes the incision and dissection and decreases the chance of injury to the new anus. The discomfort from the colostomy and anal dilation is safely avoided. Patients undergoing this approach can expect the good preservation of continence.

## Data Availability

All data generated or analyzed during this study are included in this published article.

## Abbreviations

RUF: Rectourethral fistula
ARM: Anorectal malformation
PSARP: Posterior sagittal anorectoplasty
